# Supplementary material for: A framework for multiplex imaging optimization and reproducible analysis
Source: Commun Biol. 2022 May 11;5:438. doi: 10.1038/s42003-022-03368-y (PMC9095647; doi:10.1038/s42003-022-03368-y)
Supplement: Supplementary file 3 — Description of Additional Supplementary Files [file 42003_2022_3368_MOESM3_ESM.pdf]

Description of Additional Supplementary Files

File name: Supplementary Data 1
Description: Antibodies and Experiments.
